# Designing and testing regenerative pulp treatment strategies: modeling the transdentinal transport mechanisms

**DOI:** 10.3389/fphys.2015.00257

**Published:** 2015-09-15

**Authors:** Agathoklis D. Passos, Aikaterini A. Mouza, Spiros V. Paras, Christos Gogos, Dimitrios Tziafas

**Affiliations:** ^1^Department of Chemical Engineering, Aristotle University of ThessalonikiThessaloniki, Greece; ^2^Department of Endodontology, School of Dentistry, Aristotle University of ThessalonikiThessaloniki, Greece

**Keywords:** dentinal tubule, dentin regeneration, transdentinal diffusion, bioactive molecules, CFD, μ-LIF

## Abstract

The need for simulation models to thoroughly test the inflammatory effects of dental materials and dentinogenic effects of specific signaling molecules has been well recognized in current dental research. The development of a model that simulates the transdentinal flow and the mass transfer mechanisms is of prime importance in terms of achieving the objectives of developing more effective treatment modalities in restorative dentistry. The present protocol study is part of an ongoing investigation on the development of a methodology that can calculate the transport rate of selected molecules inside a typical dentinal tubule. The transport rate of biological molecules has been investigated using a validated *CFD* code. In that framework we propose a simple algorithm that, given the type of molecules of the therapeutic agent and the maximum acceptable time for the drug concentration to attain a required value at the pulpal side of the tubules, can estimate the initial concentration to be imposed.

## Introduction

During the last decades the clinical practice has been oriented toward the design and development of modern dental treatment techniques that would ensure the long-term maintenance of vitality and function of the dentine–pulp complex (de Peralta and Nör, 2014). The traditional treatment strategies in vital pulp therapy have been mainly focused on protection of dental pulp from possible irritation presented by components released from dental materials and induction of replacement of diseased dentin by a dentin-like material (Tziafas et al., 2000). Furthermore, the fact that the pulp-dentin complex can be repaired and regenerated by forming tertiary dentin, has recently led the scientific community toward the development of novel optimal procedures and production of potential therapeutic agents for use in regenerative pulp therapies. There is a growing weight of evidence that bioactive molecules diffused through the dentinal tubules can regulate the biosynthetic activity of pulpal cells (Rutherford et al., 1995; Smith et al., 2001). In a previous experimental approach we reported that *TGF-b1* and, to a lesser extent, *BMP-7* provided evidence that stimulate tertiary dentin, while intratubular mineralization may occur when these growth factors are placed on deep dentine cavities (Kalyva et al., 2010).

Pre-clinical testing of dental materials to evaluate biologically their application includes studies in animals and humans. Human studies are most appropriate when investigating dentine-pulp reactions to various newly introduced substances and commercially available formulas. However, protection of patients from possible hazards presented by dental materials and the biological effects that they might have is needed (Murray et al., 2007). On the other hand, animals' studies have raised concerns of their capability to predict human dental pulp reactions because of the fact that there are possible species variations. Thus the numbers of both clinical and animal screening tests must be minimized to make this form of testing legally and ethically acceptable. Development of several *in vitro* approaches simulating clinical conditions that are helpful to control parameters concerning experimental conditions is in progress during the last two decades.

Parameters that have been implicated with pulpal injury, and therefore affect the pulp responses to materials placed in a dentinal cavity environment, have attracted attention. The numbers of odontoblasts surviving the injury caused by the cavity cutting and restoring procedures have been strongly correlated with cavity preparation technique and dimensions, the nature of tested materials including the diffusion rate of their components, and method of application (Smith et al., 2001). Previous data have showed that diffusion rate of dental material components exerting possible toxicity to the pulpal cells depends on the remaining dentin thickness. Murray et al. (2001); Murray et al. (2003) further reported that there is an inverse relation between the remaining dentin thickness and odontoblast survival. They concluded that an *RDT* greater or equal to 0.5 mm is necessary to protect the underlying odontoblasts form the operative trauma.

Since it is evident that a remaining dentin zone is necessary to protect the dental pulp survival and function, much has still to be learnt on the effect of potent toxic components or bioactive agents that are released from traditional and regenerative therapeutic approaches. Since it is reasonable to suggest that any toxic interruption or therapeutic up-regulation of the biosynthetic activity of odontoblasts (Smith, 2003) will probably reflect the molecular concentration of these constituents at the dentin-pulp interface area, a number of delivery considerations might be firstly addressed. Among other issues, the simulation of clinical conditions requires the development of an experimental methodology that can calculate the transport rate of selected molecules by employing advanced non-intrusive techniques. The experimental study of the diffusion rate of therapeutic molecules inside the dentin tubules is almost impossible using the established experimental techniques due to the minute dimensions of the dentinal tubules. On the other hand, the analytical solution of the governing mass and momentum transfer equations is practically infeasible due to the non-uniformity of the tubules cross section. Therefore, Computational Fluid Dynamics (*CFD*) seems to be the only feasible method for studying the problem under consideration. Recent studies confirmed that *CFD* is a powerful analytical tool in dental pulp research (Boutsioukis et al., 2009; Gao et al., 2009; Su et al., 2014). However, an up to date literature review has revealed that there is no significant progress interpretation of flow phenomena inside the dentinal tubules. It is, therefore, desirable to be able to suggest a new integrated computational approach that could adequately simulate both the fluid flow and diffusion characteristics inside the tubules.

The idea of using the dentinal tubules for drug transfusion in pulp was originally proposed by Pashley (1990), but there is limited information on the balance between the rate of internal diffusion of exogenous factors (e.g., drugs, molecules, bacterial byproducts) and the resistance of outward fluid flow inside a dentinal tubule. Later Pashley (1992a,b) suggested that the therapeutic agents must consist of small molecules or ions that exhibit relatively high diffusion coefficients, while high concentrations must be avoided due to the risk of increasing the rate of movement of dentinal fluid outwardly and thus prohibiting the internal diffusion of the drug. According to the dental clinical practice the resistance to the internal diffusion due to the fluid outflow could be considered to be minimal, if the application of the capping agent is followed by immediate covering the exposed dentinal surface with an adhesive dental material. It has been previously documented that large molecules such as endotoxins (1–30 kDa) or exotoxins (20–70 kDa) can dissolve in the dentinal fluid and diffuse to the pulp (Pashley and Matthews, 1993).

The present work is part of an ongoing investigation on phenomena related to the dental therapeutic practice and is expected to provide insights on the key issues of transdentinal regulation of the dentin-pulp complex functions. This article comprises the study of the diffusion of substances similar to dental materials components and bioactive agents used in today clinical practice and promising regenerative approaches respectively. Therefore, we describe the basic principles for the development of a model that would simulate the flow and mass transfer in the dentinal tubules and propose a numerical methodology for investigating the mass transfer characteristics in a microtube (μ-tube) that closely resembles a typical dentinal tubule geometry. The *CFD* code is validated using the outcome of an analytical solution as well as with experimental data acquired using the non-intrusive experimental technique μ*-LIF*.

The *CFD* simulations concerning mass transfer of chemical compounds confirm that:

- the diffusion is a very slow process, a fact verified by clinical observations,
- large molecules penetrate at a lower rate inside the dentinal tubules compared to smaller ones,
- the mass flux inside a tubule increases by increasing the initial concentration of the substance and
- the time required for the concentration of the signaling molecules to attain a *predefined* value at the pulp can be controlled by their *initial* concentration.

Thus, in the framework of developing more effective treatment modalities in restorative dentistry we are able to provide an algorithm that, given:

- the type, i.e., the size of the molecules of the therapeutic agent,
- the required drug concentration at the pulpal side of the tubules and
- the maximum *acceptable time* to attain the aforementioned concentration,

can estimate the *initial concentration* that must be imposed.

## Materials and methods

The commercial *CFD* code *ANSYS CFX*® *15.0* is employed for the simulations, i.e., for solving the incompressible *Navier-Stokes* and mass transfer equations (McCabe et al., 2005). This paper tackles the problem of the diffusion of substances through a conical μ-tube that simulates a typical dentinal tubule.

However, before proceeding to the computational simulations, is common practice the *CFD* code to be validated. As already mentioned, the theoretical and experimental study of the diffusion process inside the dentinal tubules is practically impossible. Therefore, at an initial stage the flow through a *cylindrical* μ-channel studied using the *CFD* code and the numerical results are compared with the analytical solution of *Fick's Second Law*. Experiments concerning the diffusion of a fluorescent dye in water are also performed in a glass capillary. The local dye concentration is measured by the non-intrusive μ-*LIF* measuring technique and the acquired experimental data are compared with the *CFD* results. It is therefore quite reasonable to assume that the validated computational code can be applied for studying the diffusion of substances similar to potential therapeutic agents through a typical dentinal tubule. In this stage and in accordance with the dental clinic practice, *no fluid outflow* from the μ-tube is assumed.

**Figure 1:**
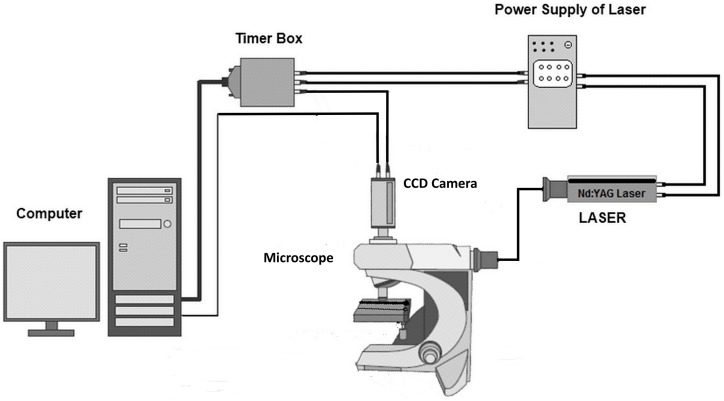
**μ-LIF experimental setup**.

As already mentioned, for the experimental study of the diffusion in μ-channels and for validating the *CFD* code, the non-intrusive micro Laser Induced Fluorescence (μ*-LIF*) technique was employed. Laser-Induced Fluorescence (*LIF*) is an optical measuring technique used to measure instant whole-field concentration maps in liquid and gaseous flows. This can be done indirectly by measuring the light emitted (i.e., fluorescence) by the tracer compounds that are used to mark the fluids. The *LIF* experimental setup, available in our Lab, is shown in Figure 1. The experiments were performed in a glass capillary (*ID* = 580μm, *L* = 3 cm). The measuring section was illuminated by a double cavity *Nd:YAG* Laser emitting at 532 nm. The fluid flux was measured by a high sensitivity *CCD* camera (*Hisense MkII*), connected to a Nikon (*Eclipse LV150*) microscope, which moves along the vertical axis with an accuracy of one micron. A *10X* air immersion objective with *NA* = 0.20 was used. For each measurement at least 20 images were acquired at a sampling rate of 5 Hz.

The experiments were performed using two liquids, namely distilled water as reference and distilled water marked with the fluorescent dye *Rhodamine B*, completely dissolved in water, whose diffusion coefficient in water is 4.5·10^−10^ m^2^/s (Gendron et al., 2008). *Rhodamine B* is a fluorescent dye with a quantum yield of up to φ = 0.97 at low concentrations. As reported by Bindhu and Harilal (2001) its quantum yield depends on the solvent and on the type of excitation (continuous, pulsed) and decreases weakly with increasing concentration. *Rhodamine B*, dissolved in water, is most effectively excited by green light and emits red light with the maximum intensity in the range 575–585 nm (Sakakibara and Adrian, 1999; Bindhu and Harilal, 2001). Image processing and concentration calculations were performed using appropriate software (*Flow Manager* by *Dantec Dynamics*).

A typical procedure for μ-LIF measurements consists of the following steps:

- Determination of the *relationship* between the measured fluorescence intensity field *I*_(*x, t*)_ and the concentration field *C*_(*x, y*)_.
- Calibration experiments prior to the actual measurements using a series of aqueous *Rhodamine B* solutions with known concentrations, namely *C* = 0.05, 0.025, and 0.00 mg/L. For each concentration *C*_*i*_ an image *C*_(*x, y*)_ is taken.
- Image masking of the acquired images is also necessary in order to reduce noise and then an appropriate *Region of Interest* (*ROI*) is defined, at which the fluorescence intensity is measured.

For our experiments the optical system was set to have a *ROI* of 300*x*580 μm, while the lens was focused on the middle plane of the capillary. Care was taken to ensure that the experiments are conducted at identical conditions with the calibration procedure, so as to minimize the uncertainty of the experimental procedure.

**Figure 2:**
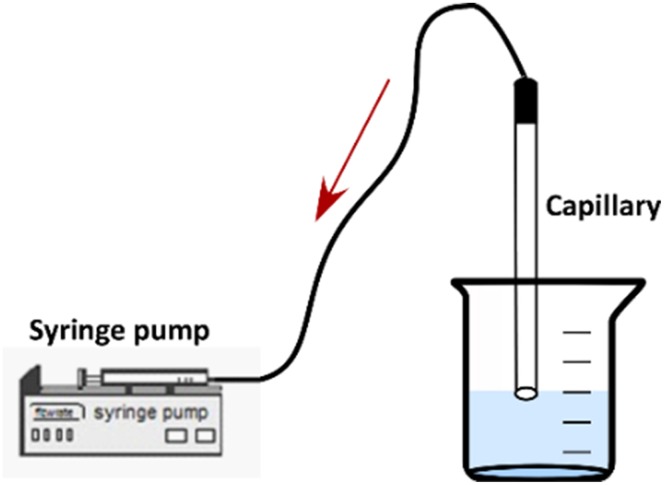
**Test-section connected with a syringe pump**.

After the calibration of the measuring system, the two liquids, namely water and the colored aqueous solution at *C* = 0.05 mg/L, were successively inserted into the capillary. A syringe pump (*ALLADIN 2000*) was employed for the suction of the two liquids inside the capillary (Figure 2).

It is very important that the fluids enter the capillary at a very low velocity so as to avoid mixing due to convection, although a small initial concentration distribution of *Rhodamine B* inside the capillary is unavoidable. After the two liquids have entered the μ-channel, the syringe pump is turned off and the capillary is placed carefully under the microscope at a horizontal position for conducting the *LIF* measurements. The test-section remains connected to the syringe pump during the measurements to ensure constant pressure condition inside the μ-channel.

Finally, concentration measurements were performed at 10–15 locations on the middle plane, at 1 mm from each other along the axis of symmetry of the μ-tube. As there is no lateral diffusion, i.e., the process is *1D*, we can assume that the concentration is constant along the diameter of the μ-tube. Thus the *LIF* measurement corresponds to the concentration at a cross-section of the conduit. The axial length *x* is normalized with respect to the tube length, *L*; thus *x/L* = *0* denotes the entrance of the capillary.

The image processing of the *LIF* images comprises the definition of the mean image out of the set of 20 images acquired and their processing according to the *LIF* calibration record that has been created. After the experimental measurements, appropriate numerical simulations that fully represent the initial experimental conditions are performed.

**Figure 3:**
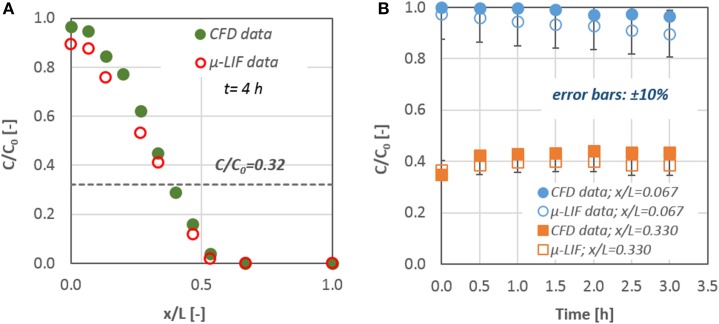
**Comparison of CFD data with the corresponding results from the LIF technique: (A) mass concentration distribution inside the capillary after *t* = 4 h and (B) mass concentration values for x/L = 0.067 and 0.330; error bars: ±10%**.

In Figure 3A a typical comparison of *CFD* data with the corresponding results from the μ*-LIF* technique is presented concerning the concentration inside the channel after *t* = 4 h. The dashed line represents the calculated final concentration value *C*_*t*_ inside the capillary for *t* = 8. In Figure 3B the two methods are compared with respect to the temporal concentration variation at two locations inside the capillary (*x/L* = 0.067 and 0.330).

The experimental results are in fairly good agreement (< 10%) with the *CFD* data. The discrepancy between the two methods may be attributed to the fact that the experimental data concern the mean concentration values that were measured at the applied *ROI*, while the corresponding values from the *CFD* results give the concentration at the center of a grid cell with an axial dimension of 5 μm. After comparing the data of the numerical simulations with the available experimental results from the μ*-LIF* technique we can come to the conclusion that the *CFD* code is able to predict diffusion of substances through liquids in such small diameter conduits.

### Theoretical approach

The ability of the computational code to effectively simulate diffusional mass transfer in μ-conduits is confirmed by comparing the *CFD* results with analytical solutions of the governing equations for molecular mass transport. Diffusion is the net movement of a substance from a region of high concentration to a region of low concentration, i.e., it is the movement of a substance down a concentration gradient. In steady state conditions the diffusive flux *j* of a substance driven by the concentration gradient in one dimension is given by the Fick's First Law (Equation 1):

(1) $$\begin{array}{l} j=-D\frac{\partial C}{\partial x} \end{array}$$

where *j* is the flux, *D* is a diffusion coefficient, ∂*C*∕∂*x* is the concentration gradient and *x* is the distance in the direction of transfer. This equation follows the general rule that matter diffuses from a region of higher concentration to a region of lower concentration, hence the minus sign. The diffusion coefficient *D* is a function of temperature, and its units are in length squared per unit time, provided that *j, C* and *x* are in consistent units.

Fick's Second law is used to predict how diffusion causes the concentration to change with time, i.e., unsteady conditions, and in one dimension is given by Equation (2):

(2) $$\begin{array}{l} \frac{\partial C}{\partial t}=D\frac{\partial^{2}C}{\partial x^{2}} \end{array}$$

where *t* is the time, *C* the concentration which is a function of both location *x* and time *t*.

To comply with the actual case of transdentinal diffusion of substances, there is need to apply a rigorous analytical equation, originated from the Fick's Second Law, that incorporates a zero flux boundary condition at *x/L* = 1, i.e., the pulpal area. The new formulated analytical solution which is a sum of a cosine series is given by Equation (3):

(3) $$\begin{array}{l} C_{\left(\chi ,t\right)}=\frac{2M}{L}\overset{\infty }{\underset{n=1}{\Sigma }}e^{-{\left(n\pi ∕L\right)}^{2}tD}\cos\left(n\pi x∕L\right)+M_{t} \end{array}$$

where, *M* is the total amount per area of the diffusing species, *x* the distance from the source, *L* the channel length and *M*_*t*_ the final estimated concentration value inside the μ-channel.

**Figure 4:**
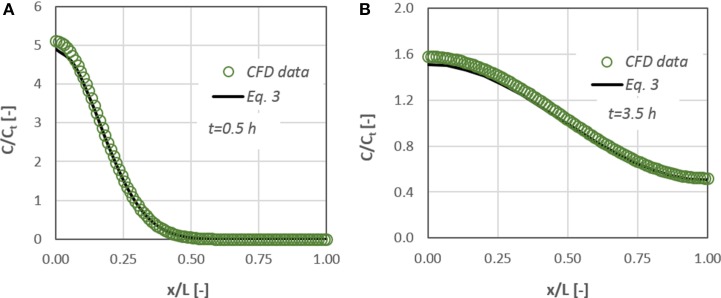
**Concentration distribution of a diffusate in a cylindrical μ-tube**. Comparison between CFD data and the corresponding results from Equation (3) for: **(A)** *t* = 0.5 h and **(B)** *t* = 3.5 h after the diffusate release.

Figure 4 presents the comparison between the results of Equation (3) and the data obtained from the *CFD* simulations for two time periods (i.e., 0.5 and 3.5 h). The initial mass of the diffusate is located at *x/L* = 0. A very good agreement (±10%) exists between the experiments and the analytical solution with the corresponding numerical ones. Consequently, we can conclude that the *CFD* code is capable of handling diffusion and mass transport phenomena in the microscale.

### Numerical procedure

For studying the transdentinal diffusion, we use substances whose molecular sizes are the same as those of potential therapeutic agents that are used in dental clinic practice. The radius *R*_*min*_ and the diffusion coefficient *D* of a protein can be determined by Equations (4, 5) (Erickson, 2009).

(4) $$\begin{array}{l} R_{\min}=0.066M_{w}^{1∕3} \end{array}$$

where *R*_*min*_ is given in nanometers and the molecular weight *M*_*w*_ in Daltons (*Da*) and

(5) $$\begin{array}{l} D=\frac{kT}{6\pi \mu R_{s}} \end{array}$$

where *k* = 1.38 × 10^−16^ g cm^2^ s^−2^ K^−1^ is the *Boltzman's* constant and *T* the absolute temperature. *k* is given here in centimeter–gram–second units because *D* is expressed in centimeter–gram–second, while μ that stands for the solute viscosity is in g/(cm·s). *R*_*s*_, called *Stokes* radius, represents the radius of a smooth sphere that would have the same frictional coefficient *f* with a protein and is expressed in centimeters in this equation. Assuming that *f* equals *f*_*min*_, i.e., the minimal frictional coefficient that a protein of a given mass would obtain if the protein was a smooth sphere of radius *R*_*min*_, then *R*_*min*_ could replace *R*_*s*_ in Equation (5).

In Table 1 some representative examples of substances or ions released from dental materials in use or experimentally designed bioactive agents are presented. Released monomers from resins (*TEGDMA*) or dental adhesives (*HEMA*) disturb cell functions including responses of the immune system, mineralization, and differentiation of dental pulp cells, or induce cell death via apoptosis (Schweikl et al., 2014). The role of bases and liners (zinc oxide-eugenol or calcium hydroxide or calcium silicate-based materials) indicated for pulp protection is still controversial in terms of molecules or ions diffusion through the exposed dentinal tubules. Furthermore biologically active molecules applied as possible therapeutic drugs with an appropriate delivery system (*BMP*-7) or endogenous growth factors released after dentin treatment with *EDTA* (*TGFbeta*) have shown important tissue specific activity for dentin-pulp complex regeneration.

**Table 1:** **Representative traditional or experimental applications in the situation of exposed dentinal surfaces with the characteristics of released molecules or ions**.

**Material group for dental use**	**Released molecules/Ions**	**Molecular formula/Characteristics**	**Molecular/Atomic weight (Da)**	**Diffusion coefficient (m^2^/s)**
Composite resins-tooth restoration	TEGDMA Triethylene glycol dimethacrylate	CH_2_ = C(CH_3_)COO(CH_2_CH_2_O)_3_COC(CH_3_) = CH_2_	286	7.5·10^−10^
Bonding agents-tooth restoration	HEMA 2-Hydroxyethyl methacrylate	CH_2_ = C(CH_3_)COOCH_2_CH_2_OH Polymer absorbing up to 600% water. Basically it is hydrophobic but contains hydrophilic group.	130	9.7·10^−10^
Glass ionomer cements- pulp protection	Fluoride ions	F^−^	9	2.4·10^−09^
Zinc oxide-eugenol bases-pulp protection	Eugenol 4-Allyl-2-methoxyphenol	C_10_H_12_O_2_hydrophobic	164	8.9·10^−10^
Calcium hydroxide-based liners-pulp protection	Calcium ions	Ca^++^	20	1.8·10^−09^
Calcium silicate-based bases -pulp protection	Silicon	Si^4−^	14	2.1·10^−09^
EDTA-soluble dentinal constituents (experimental applications) Pulp protection	Transforming growth factor -beta	Polypeptide	44,300	1.4·10^−10^
BMP7-containing capping agents (experimental applications) pulp protection	Bone morphogenetic protein 7	Polypeptide	50,000	1.3·10^−10^

In this computational study *three different substances* were used. The radius *R*_*s*_ of their molecules is in the range of 2.2–22.0 nm and consequently the corresponding diffusion coefficient, *D*, in water at 37 C is in the range of 1.36–0.14·10^−10^ m^2^/s. The range of molecular sizes employed in this study corresponds to the size of actual bioactive molecules. For example, *BMP*-7 is such a bioactive protein used in the dental clinic practice with approximately 50 kDa molecular weight and *R*_*s*_ of 2.2 nm. To conduct a parametric study, three initial concentration values for each diffusing substance are also employed, namely 0.10, 0.05, and 0.01 mg/mL.

We studied the effect of:

- the initial diffusate concentration and
- the molecular size

on the transdentinal diffusion characteristics.

A grid dependence study has been also performed for the case involving the diffusate with the highest diffusion coefficient value. As the study comprises the time-dependent solution of diffusion, all simulations were run in *transient mode*. A time-step dependence study was also performed, to ensure that a suitable time step is selected for each simulation, i.e., the minimum number of calculation steps performed without jeopardizing the accuracy of the solution. The total simulation time for each run was set at 10 h as the diffusion process is expected to be extremely slow.

## Anticipated results

**Figure 5:**
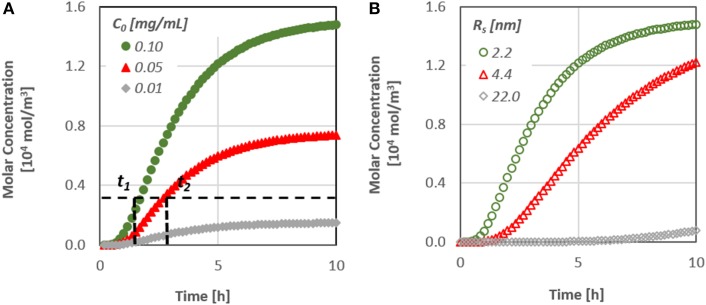
**(A)** Effect of the initial substance concentration on the diffusion characteristics [*t*_(1, 2)_ time needed to reach a specified concentration value at the pulp] and **(B)** Effect of the molecular size on the diffusion characteristics.

The diffusion of a substance similar to the therapeutic agent *BMP-7*, through a dentinal tubule under different values of initial concentration has been studied. Figure 5A, which presents the therapeutic agent concentration at the bottom of the dentinal tubule, i.e., at the dentin-pulp junction, as a function of time, demonstrates the effect of initial substance concentration on the concentration change. Figure 5B shows the effect of the type of substance, i.e., its molecular size, on the concentration change at the pulpal area.

It is obvious that the diffusion rate is particularly low, since it takes more than 10 h for the concentration at the pulpal side to reach its final value, although after approximately 2 h, the first molecules of the substances do reach the bottom end. In addition, as the diffusion coefficient is a function of the size of the molecules, substances/proteins with larger molecules penetrate at a lower rate inside the dentinal tubules.

It is worth noting that the time required for the concentration of signaling molecules to attain a specific value at the pulp can be controlled by their initial concentration. By increasing the initial concentration of a potential therapeutic agent, the mass flux inside the dentinal tubule increases and as a result the signaling time of the molecules may also decrease. This is an important finding, especially for the dental community, since it gives the possibility to predict the behavior of each therapeutic agent prior to its application on the dental clinic practice.

Obviously it is important to be able to predict the final concentration of an applied therapeutic agent. In this case one must run the relevant *CFD* simulation to produce an appropriate curve like the one presented in Figure 5. However, this is not a trivial procedure to be used in every day practice. Thus, an effort was made to propose a simple correlation that could predict with reasonable accuracy the temporal concentration value at the pulp through the dentinal tubules, as a function of the diffusion coefficient of the substance, i.e., its molecular size. The proposed correlation can be then used to estimate the maximum required time for the diffusion process to reach steady state condition, i.e., when the concentration of the substance has the same value along the tubule, after calculating the term *L*^2^*/D*. The proposed correlation, given in Equation (6), has been formulated by data fitting on the computational results and incorporates the dimensionless time t^*^:

(6) $$\begin{array}{rcl} C∕C_{t} & = & \exp\left(0.21+\frac{0.115}{t^{*}}\cdot \ln\;\left(t^{*}\right)\right) \end{array}$$

(7) $$\begin{array}{rcl} \text{where }t^{*} & = & Dt∕L^{2}. \end{array}$$

**Figure 6:**
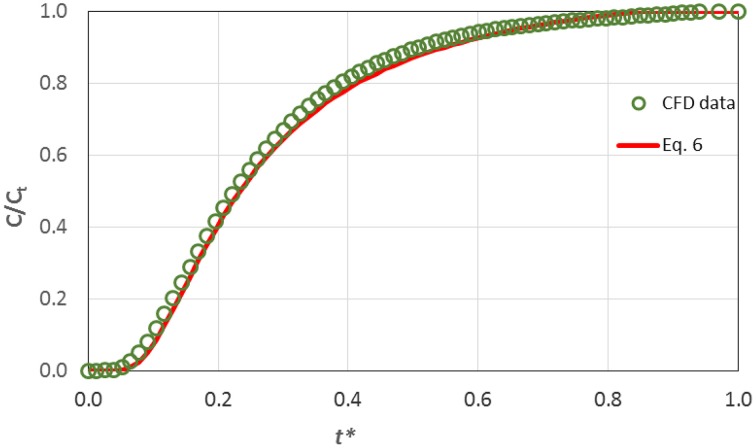
**Comparison of CFD results on the transdentinal diffusion of a protein (R_*s*_ = 2.2 nm, C_0_ = 0.10 mg/mL) with the prediction of Equation (6)**.

In Figure 6 the computational results concerning the dimensionless concentration at the pulpal area vs. the dimensionless time t^*^ are compared with the values predicted by Equation (6).

The data are in excellent agreement with the prediction of the proposed correlation and consequently one can draw the conclusion that Equation (6) is suitable for estimating the concentration at the pulp during the diffusion process. Obviously, the proposed correlation can be applied for substances with various molecular sizes *R*_*s*_ and hence different diffusion coefficients, since diffusion in dentinal tubules can be safely considered one-dimensional.

## Stepwise procedure

**Figure 7:**
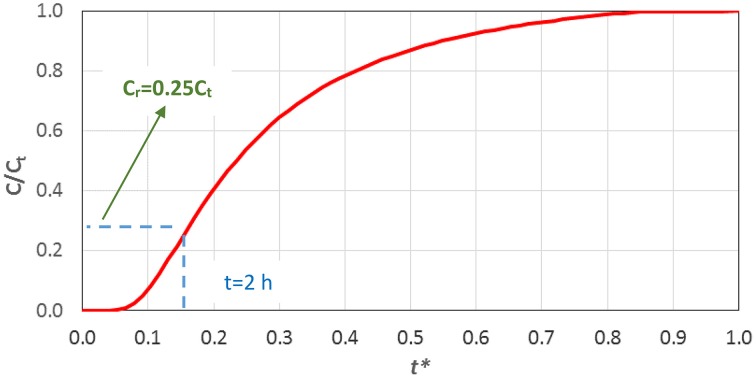
**Graphical solution of Equation (6)**.

In the present protocol study a stepwise procedure for estimating the diffusate concentration to be imposed at the dentin-enamel junction so as the drug concentration to attain a *required value* at the pulpal side of the tubules is proposed given the geometrical characteristics of the dentinal tubules:

- Estimate the diffusion coefficient of the agent through Equations (4, 5), given its size or molecular weight.
- Choose the required value of the drug concentration, *C*_*r*_, at the pulpal side of the tubules.
- Choose the maximum acceptable time, *t*, for the drug concentration to attain the required value *C*_*r*_.
- Calculate the initial concentration *C*_0_ of the substance by solving Equation (6) either numerically or graphically (Figure 7).

A detailed example concerning the application of this stepwise procedure, based on Figure 7, for the estimation of the initial concentration of the therapeutic agent (*C*_*o*_), in order to achieve the required final concentration (*C*_*r*_) after an acceptable time (*t*) is given hereafter.

- A typical protein with diameter *R*_*s*_ = 2.2 nm is considered.
- The diffusion coefficient *D* of the protein calculated by Equation (5) is 1.36·10^−10^ m^2^/s.
- A typical length (*L*) of a dentinal tubule can be considered to be 2.5 mm.
- Using the aforementioned values and Equation (6) one can plot the corresponding curve (Figure 7).
- If it is desirable for the required drug concentration value to reach the pulp within 2 h, the variable t^*^ = 0.15.
- From Figure 7 the ratio *C*_*r*_*/C*_*t*_ = 0.25 is determined.
- As the required effective drug concentration (*C*_*r*_) is dictated by the dental clinic practice, the final concentration (*C*_*t*_) can be calculated as *C*_*t*_ = *C*_*r*_*/*0.25.
- Finally, for a given *C*_*t*_ and the corresponding mass balance (drug quantity and tubule's dimensions one can easily estimate, from a mass balance (i.e., *C*_**0**_*V*_**0**_= *C*_*t*_*V*_*t*_, where *V*_*t*_ is the total volume of the tubulus and the drug), the initial concentration of the drug *C*_**0**_ to be imposed at the *DEJ*.

Following the stepwise procedure and assuming that *C*_*t*_ = *C*_*r*_/0.25 for the required effective concentration at the pulp for all the therapeutic substances of Table 1, the corresponding critical time are calculated through Equations (6, 7) and are presented in Table 2.

**Table 2:** **Critical time for C_*t*_ = C_*r*_/0.25 for various therapeutic agents**.

**Released molecules/Ions**	**Critical time *t_*r*_* (min)**
TEGDMA Triethylene glycol dimethacrylate	21
HEMA 2-Hydroxyethyl methacrylate	16
Fluoride ions	7
Eugenol 4-Allyl-2-methoxyphenol	17
Calcium ions	9
Silicon	8
Transforming growth factor -beta	113
Bone morphogenetic protein 7	118

## Discussion on the proposed methodology

In the present study we propose a simple algorithm which, given the type of molecules of the therapeutic agent and the maximum acceptable time for the drug concentration to attain a required value at the pulpal side of the tubules, can estimate the initial concentration to be imposed. An example on the application of the proposed methodology is given in the stepwise procedure.

We must point out here that our study deals with a sole microtube that represents a typical dentin tubule that bears no obstructions of any type. The main assumptions made are:

- The geometry comprises a typical dentinal tubule 2.5 mm long and 1 and 3 μm in diameter at the dentin-enamel and the dentin-pulp junction respectively.
- The diffusion can be safely presumed one-dimensional.
- Molecules/ions that enter a dentinal tubule do not react with each other.
- The presence of large plasma proteins underlying the pulp, such as fibrinogen (Mw >300 kDa) or immunoglobulins (150 kDa) are not expected to impede the diffusion of therapeutic agents from the *DE* junction to the pulp.

However, the overall dentin permeability may be reduced due to occlusions of one or more dentin tubules and for this reason one must be able to apply a typical value (i.e., coefficient) of potential permeability, but this is beyond the scope of the present study. In the literature several factors that may lower the dentin permeability are introduced. Pashley and Tay (2012) mention that tubules in the dentin of mature teeth can be totally occluded, partially occluded and patent due to the presence of organic fibers and inorganic crystals or that smear layer and smear plugs that are produced during operative dental treatment may cover the exposed dentinal surface and seal the dentinal tubules.

Understanding the central role that fluid dynamics plays in the maintenance of vitality and function of the dentin-pulp complex, scientists can address the problems of delivery and control of potent toxic effects of molecules released from dental materials and of biologically active molecules used in tissue engineering approaches. Both advantages offer opportunities for development of new therapies in dental clinical practice.

### Conflict of interest statement

The authors declare that the research was conducted in the absence of any commercial or financial relationships that could be construed as a potential conflict of interest.

## Figures and Tables

**Table:** 

***C***	**Molar concentration, *mol/m^*3*^***
***C_*o*_***	**Initial molar concentration, *mol/m^*3*^***
***C_*r*_***	**Required molar concentration on pulp (effective), *mol/m^*3*^***
***C_*t*_***	**Final molar concentration (for *t = L^2^/D*), *mol/m^*3*^***
***D***	**Diffusion coefficient, *m^*2*^/s***
***j***	**Concentration flux, *mol/m^2^·s***
***L***	**Length, *m***
***M***	**Mass concentration, *kg/m^*2*^***
***M_*w*_***	**Molecular weight, *g/mol***
***R_*s*_***	**Stokes radius, *m***
***r***	**Radius of conduit, *m***
***T***	**Temperature, *C***
***t***	**Time, *s***
t^*^	***Dt/L^*2*^, dimensionless***
***u***	**Velocity, *m/s***
***x***	**Distance, *m***
**μ**	**Viscosity, *g/cm·s***
**ρ**	**Density, *kg/m^*3*^***
